# Reboxetine Treatment Reduces Hippocampal Gliosis in the P301S Tauopathy Mouse Model

**DOI:** 10.1080/17590914.2026.2630485

**Published:** 2026-02-21

**Authors:** Irene L. Gutiérrez, Claudia Yanes-Castilla, Karina S. MacDowell, Javier R. Caso, Borja García-Bueno, Cristina Ulecia-Morón, Juan C. Leza, José L. M. Madrigal

**Affiliations:** a Department of Pharmacology and Toxicology, School of Medicine, Universidad Complutense de Madrid (UCM), Madrid, Spain; b Instituto de Investigación Neuroquímica (IUIN-UCM), Instituto de Investigación Sanitaria Hospital, Madrid, Spain; c Centro de Investigación Biomédica en Red de Salud Mental, Instituto de Salud Carlos III (CIBERSAM, ISCIII), Madrid, Spain; d Institut d’Investigacions Biomèdiques de Barcelona (IIBB), Spanish National Research Council (CSIC), Barcelona, Spain;; e Institut d’Investigacions Biomèdiques August Pi i Sunyer (IDIBAPS), Barcelona, Spain;; f Department of Molecular Biology, Universidad Autónoma de Madrid (UAM), Centro de Biología Molecular Severo Ochoa (CBM-CSIC), Instituto Universitario de Biología Molecular (IUBM), Madrid, Spain

**Keywords:** Alzheimer, neuroinflammation, P301S, reboxetine, tau

## Abstract

The loss of brain noradrenergic neurons is one of the earliest alterations observed in Alzheimer’s disease and other neurodegenerative pathologies. The consequent reduction of brain noradrenaline levels facilitates the progression of neuroinflammatory processes that can be fatal for neurons and other brain cells. For this reason, compensating for noradrenaline deficit through different means constitutes an interesting therapeutic strategy. Drugs that inhibit the reuptake of noradrenaline are used to elevate the extracellular concentrations of this neurotransmitter and potentiate this way its effects. These drugs are approved for the treatment of depression or attention deficit hyperactivity disorder, among other indications, but their repurposing and use in Alzheimer’s disease could be of interest given the beneficial effects observed for noradrenaline in numerous studies. Based on this, we previously showed the beneficial effects of reboxetine, a noradrenaline reuptake inhibitor, on 5xFAD mice that accumulate amyloid beta in their brains and reproduce some of the typical alterations of Alzheimer’s disease. In this study we have analyzed the effects of reboxetine on P301S mice, a different model of Alzheimer’s disease based on the expression of mutant forms of human microtubule-associated protein tau. We observed that the administration of reboxetine with osmotic pumps for 28 days to 9-month-old mice reduced the accumulation and activation of microglia and astrocytes in different areas of the hippocampus. These findings indicate that reboxetine treatment prevents the neuroinflammatory response known to cause brain damage in Alzheimer’s disease even when the treatment is initiated at an advanced stage of the disease.

## GRAPHICAL ABSTRACT

**Figure:**
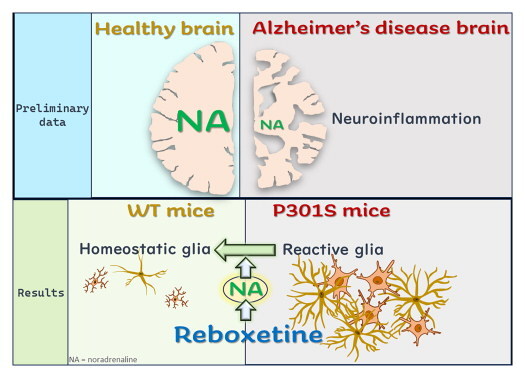

## Introduction

One of the best characterized alterations occurring during the first stages of Alzheimer’s disease (AD) is the loss of noradrenergic neurons (Gannon et al., 2015). These cells are grouped in small brainstem nuclei in the pons and lateral reticular formation of the medulla. Among these nuclei stands out the *locus coeruleus* (LC) as it seems to be the largest one and the main source of noradrenergic innervation (Szabadi, 2013). The LC also seems to be the noradrenergic nucleus most damaged in AD and, consequently, the most studied one for its potential relevance as a target for anti-neurodegenerative treatments (Chen et al., 2022). Numerous *in vitro* and *in vivo* studies demonstrate that strategies aimed at preventing the loss of noradrenergic neurons or compensating for the lack of endogenous noradrenaline can reduce some of AD characteristic alterations including memory loss, neuronal decay and neuroinflammation (Gutiérrez et al., 2022). This way, our previous analyses allowed us to observe how the direct treatment of cultured glial cells with noradrenaline prevented their activation in response to different kinds of stimuli, as well as the associated damage to neurons normally occurring as a result of microglia and astrocytic production of certain cytotoxic agents (Braun et al., 2014). Based on this, we have also analyzed whether different products capable of increasing noradrenaline levels in the brain could exert a protective effect in AD (Gutiérrez et al., 2019; Hinojosa et al., 2011). Some of these drugs are currently approved as medication for the treatment of different pathologies, such as depression or attention deficit hyperactivity disorder, in which an elevation of the extraneuronal concentration of noradrenaline is the main intended effect (Brunello et al., 2002). Therefore, considering the previously reported neuroprotective effects of these drugs, it seems reasonable to study their potential as alternative or complementary treatment in AD or other neurodegenerative disorders. Considering this, we have previously tested the effects of reboxetine, a selective inhibitor of noradrenaline reuptake, in the 5xFAD murine model of AD based on the overexpression of human amyloid precursor protein (APP). This allowed us to demonstrate that reboxetine treatment reduces the neuroinflammation and subsequent neuronal loss caused by the excessive accumulation of amyloid β protein that takes place in the brains of 5xFAD mice (Gutiérrez et al., 2019). However, while the altered processing of APP is undoubtedly one of the most relevant perturbations associated to AD, the other hallmark alteration of this disease is the accumulation of intraneuronal neurofibrillary tangles composed of hyperphosphorylated tau aggregates (Bloom, 2014). Therefore, we decided to further analyze the therapeutic potential of reboxetine in a different mouse model based on the expression of the P301S mutant form of human microtubule-associated protein tau (*MAPT*), as these animals closely reproduce the alterations due to tauopathies found in AD (Yoshiyama et al., 2007). The use of this model allowed us to study the effects of reboxetine treatment on the alterations caused exclusively by the accumulation of hyperphosphorylated tau and complement the previous studies performed on 5xFAD mice.

For this work we used 9 months old P301S mice, an age at which most of the symptoms of AD attributable to the accumulation of tau are detectable. The analyses performed on hippocampal samples allowed us to observe that the administration of reboxetine reduced the accumulation of microglia and astrocytes in this brain area. In addition, the morphological alterations of microglia and astrocytes normally found in P301S mice were also reduced by reboxetine treatment, confirming the anti-inflammatory effect of reboxetine as these changes are associated with a more reactive glial phenotype.

## Methods

### Mouse Models

All experimental protocols adhered to the guidelines of the Animal Welfare Committee of the Universidad Complutense of Madrid, Spain (PROEX 159.2/23), and according to European Union laws (2010/63/EU).

Male Wild-type (WT) C57BL/6 and Tau P301S (B6;C3-Tg(Prnp-MAPT*P301S) PS19Vle/J) mice were obtained from The Jackson Laboratory. These mice were maintained for over 10 generations on a C57Bl6 background.

P301S mice express the P301S mutant form of human microtubule-associated protein tau (*MAPT*), which is hyperphosphorylated and begins to accumulate in the brain after 5 months approximately.

For these studies, 9-month-old male WT and heterozygous P301S mice were used. Six or more mice were included in each experimental group. Mice were housed up to 4 per cage in a controlled 12:12 h light/dark cycle, with food and water provided ad libitum. All efforts were made to minimize animal suffering and to reduce the number of animals used.

### Reboxetine Treatment

Alzet^®^ osmotic minipumps (model 2004, delivering 0.25 μL/h for at least 28 days) were loaded with saline serum (vehicle) or with filter-sterilized reboxetine mesylate (Abcam ab120157, *t*_1/2_ = 12.5h; IC_50_ 5-HT/NE = 130) dissolved (50 mg/mL) in saline serum and kept submerged in isoosmolar saline solution at 37 °C for approximately 2 h to initiate a steady flow delivering at an approximate dose of 10 mg/kg of body weight each day.

At 9 months of age, all the mice were anesthetized by inhalation of isofluorane (induced in a chamber at 4% and maintained at 2% using a nose cone), a small incision was made behind the neck, the pumps were implanted subcutaneously between the scapulae and the incision was closed with sutures. At the end of the procedure, the surgical site was scrubbed with a 100 mg/mL povidone-iodine solution. Body temperature was maintained at 37 °C during surgery and until animals were ambulatory. Mice were monitored 2 hours after surgery and once daily until the end of the treatment. During this time, incision healing, weight loss, activity level, posture, fur appearance, and eye squinting were evaluated. If an alteration would have been observed in two of the symptoms inspected or more than a 20% weight loss would have been detected, mice would have been euthanized by cervical dislocation. However, none of the mice used in this study was euthanized before the end of the treatment due to apparent pain, distress, or discomfort.

At 29 days after the implantation of the pumps, the animals received terminal anesthesia using sodium pentobarbital (250 mg/kg i.p. Vetoquinol^®^) and subjected to transcardial perfusion with saline. The brains were removed, one hemibrain was dissected, cortical and hippocampal areas excised from the brain, frozen and kept at −80 °C. The other half was post-fixed in 4% paraformaldehyde overnight and cryoprotected in 15% sucrose during the following 24 h. After this, regularly spaced series of 25 μm-thick coronal sections were collected in cryoprotectant solution and stored at −20 °C in 24-well plates until processing.

### mRNA Analysis

Total cytoplasmic RNA from tissues was prepared using TRIzol^®^ reagent (Thermo Fisher Scientific), aliquots converted to cDNA using random hexamer primers and SuperScript^®^ Reverse transcriptase (Thermo Fisher Scientific), and mRNA levels estimated by quantitative real-time PCR (QPCR). PCR conditions were 35 cycles at 95 °C for 10 s, annealing at 60 °C for 15 s, and extension at 72 °C for 30s followed by 5 min at 72 °C in a Corbett Rotorgene. Reactions were carried out in the presence of Sybr^®^ Green (Biotools). Relative mRNA levels were calculated by comparison of take-off cycles and normalized to values for GAPDH measured in the same sample. The primers used (all listed 5′ to 3′) were

GFAP f: AAG GTC TAT TCC TGG CTG CAC AGT
GFAP r: AGC TTG GAG AGC AAC AGC TAG TCA
CX3CR1 f: GGA GAC TGG AGC CAA CAG AG
CX3CR1 r: TCT TGT CTG GCT GTG TCC TG
S100B f: GAG GAC TCC AGC AGC AAA GG
S100B r: CAC CAC TTC CTG CTC CTT GA
GAPDH f: TGC ACC ACC AAC TGC TTA GC
GAPDH r: GGC ATG GAC TGT GGT CAT GAG

### Western Blot

Samples were homogenized by sonication in 500 μL of PBS mixed with a protease inhibitor cocktail (Complete^®^, Roche Farma) followed by a centrifugation at 12.000 g for 15 min at 4 °C. After adjusting protein levels in the resultant supernatants, homogenates mixed with Laemmli sample buffer (Bio-Rad) and 20 μL (1 mg/mL) were loaded and the proteins size separated in 10% SDS-polyacrylamide gel electrophoresis (90 V). The proteins were then transferred to polyvinyl difluoride membranes, which were blocked with 5% milk in Tris-buffered saline containing 0.1% Tween-20 for 1 h and incubated overnight at 4 °C with primary antibodies against GFAP (ab4678, Abcam) or β-actin (A5441, Sigma). This was followed by incubation with anti-IgG-horseradish peroxidase-labeled secondary antibodies for 1 h at room temperature and subsequent detection with an enhanced chemiluminescence detection kit (ECL, Amersham Life Science). Blots were imaged using an Odyssey^®^ Fc System (Li-COR Biosciences). Several exposition times were analyzed to ensure the linearity of the band intensities. The resulting bands were quantified by densitometry using ImageJ Fiji^®^ analysis software (National Institutes of Health, Bethesda, MD, USA). All densitometries are expressed in arbitrary units (AU).

### Immunohistochemistry

Brain sections were washed with PBS for 5 minutes and blocked with 10% normal serum and 0.2% Triton X-100^®^ in PBS at 25 °C for 1 hour. Next, they were incubated with primary antibodies for GFAP (Abcam ab4674) and Iba1 (Wako 019–19741) diluted in 1% normal serum and 0.2% Triton X-100 in PBS at 4 °C for 18h. After this, the sections were washed three times with PBS and incubated for 1 h at 25 °C with secondary antibodies diluted 1:500 in PBS with 1% normal serum. Then, they were washed three times for 5 min with PBS and postfixed in 3.7% paraformaldehyde in PBS for 20 min. Autofluorescence was quenched with 50 mM NH4Cl in PBS for 15 min. Finally, after washing the sections with PBS for 3 minutes, they were mounted onto the slides from PBS and then coverslipped with Fluoroshied^®^ with DAPI (Sigma).

Images were obtained on a Leica SP8 confocal microscope equipped with a Leica DFC350FX digital camera, at the CAI-UCM *Centro de Citometría y Microscopía de Fluorescencia*.

At least five sections were included in each experimental group. A square region of interest (ROI) was centered on the somatosensory area, the dentate gyrus (DG), CA1, and CA3 according to area definition by Paxinos et al. (Franklin & Paxinos, 2013).

The cortex and hippocampus (from −1 to −4mm posterior to Bregma) were analyzed, and confocal Z stacks 1 μm apart of the primary motor and somatosensory area, dentate gyrus (DG), CA1, and CA3 were obtained.

To quantify the percent immunoreactive area of GFAP^+^ astrocytes and Iba1^+^ microglia using ImageJ Fiji^®^ analysis software, raw confocal images were converted to 32-bit grayscale, the detection threshold was set manually, and the same value was applied for the quantification of all groups. The percent area occupied by the automatic threshold was recorded as the percent immunoreactive area.

The perimeter, area, soma size, and cell environmental area (CEA) were directly measured in ImageJ Fiji^®^ software. To determine the Transformation Index (TI) of microglia, each cell was manually traced, and the perimeter and area of each microglial cell were measured and the TI was calculated using the following equation: perimeter^2^/4π × area^2^ (Fujita et al., 1996).

At least two sections per mouse were analyzed for each hippocampal subregion (CA3, CA1, and DG) and the motor cortex. In each section, at least five microglial cells were analyzed. Average data were calculated for all parameters analyzed to generate one vale for each mouse.

Morphological complexity was interpreted such that lower TI values (closer to 1) correspond to more circular, ameboid-like microglia with fewer processes, while higher TI values represent more complex, highly ramified morphologies with greater perimeter-to-area ratios and smaller soma.

For astrocytic analyses, number of cells and branches length were measured following the method described by Marques et al. (Marques et al., 2023) in which the images were converted to skeletonized 8-bit images and analyzed using the Analyze Skeleton plugin from Image J.

### Flow Cytometry

Brain samples were prepared as previously described (Lee & Tansey, 2013). Briefly, mice were transcardially perfused with 0.9% NaCl, and the right hippocampus was enzymatically and mechanically dissociated in serum-free DMEM/F12 medium. Myelin and debris were removed by density centrifugation using a 35% isotonic Percoll^®^ gradient (GE Healthcare, Ref. 17-0891-01). Cell pellets were resuspended in 200 µL blocking buffer [FACS buffer (1× PBS + 5% BSA) containing anti-mouse CD16/32; BioLegend^®^, TruStain FcX^®^, Cat. #101320] and incubated for 10 min at 4 °C. Samples were then divided into two 100 µL aliquots for staining: ACSA-2–PE (Miltenyi Biotec^®^, Ref. 130-123-284) and GLAST(ACSA-1)–APC (Miltenyi Biotec^®^, Ref. 130-123-641), both at 1:50 dilution. CD11b–APC (eBioscience^™^, Ref. 17-0112-82), CD45–FITC (BioLegend^®^, Ref. 147709), and TREM2–PE (R&D Systems^®^, Ref. FAB17291P). Cells were incubated for 35 min at 4 °C protected from light, washed with 3 mL FACS buffer, centrifuged (900 g, 6 min, 10 °C), and resuspended in 200 µL FACS buffer containing 100 µL DAPI (for dead-cell exclusion). An appropriate negative control was used for each staining procedure to identify each antibody.

Data were acquired on a CytoFLEX^®^ flow cytometer (Beckman Coulter). Voltages and compensations were optimized using single-stained controls to minimize spectral overlap. Data were analyzed using FlowJo^®^ (BD Biosciences) software v2.0. Cells were first gated by size and granularity (FSC-H vs SSC-H) to exclude debris, followed by singlet selection (FSC-A vs FSC-H) and live-cell gating (DAPI^-^). Astrocytes were identified as ACSA-2^+^ cells, and the mean fluorescence intensity (MFI) of ACSA-2 was quantified. Microglia were defined as CD11b^+^/CD45^Int^, and MFI for TREM2 was determined within this population.

### Statistical Analysis

All analyses were conducted using GraphPad Prism 8.0.1 and a *P* value < 0.05 was considered statistically significant.

All experiments were undertaken at least in triplicate. Data were analyzed by two-way analysis of variance (ANOVA) analyses followed by Sidak′s post hoc comparisons, using the treatment type as the first variable and genotype as the second variable.

## Results

### Reboxetine Treatment Reduces the Density of Microglia in the Hippocampus of P301S Mice

**Figure 1.**
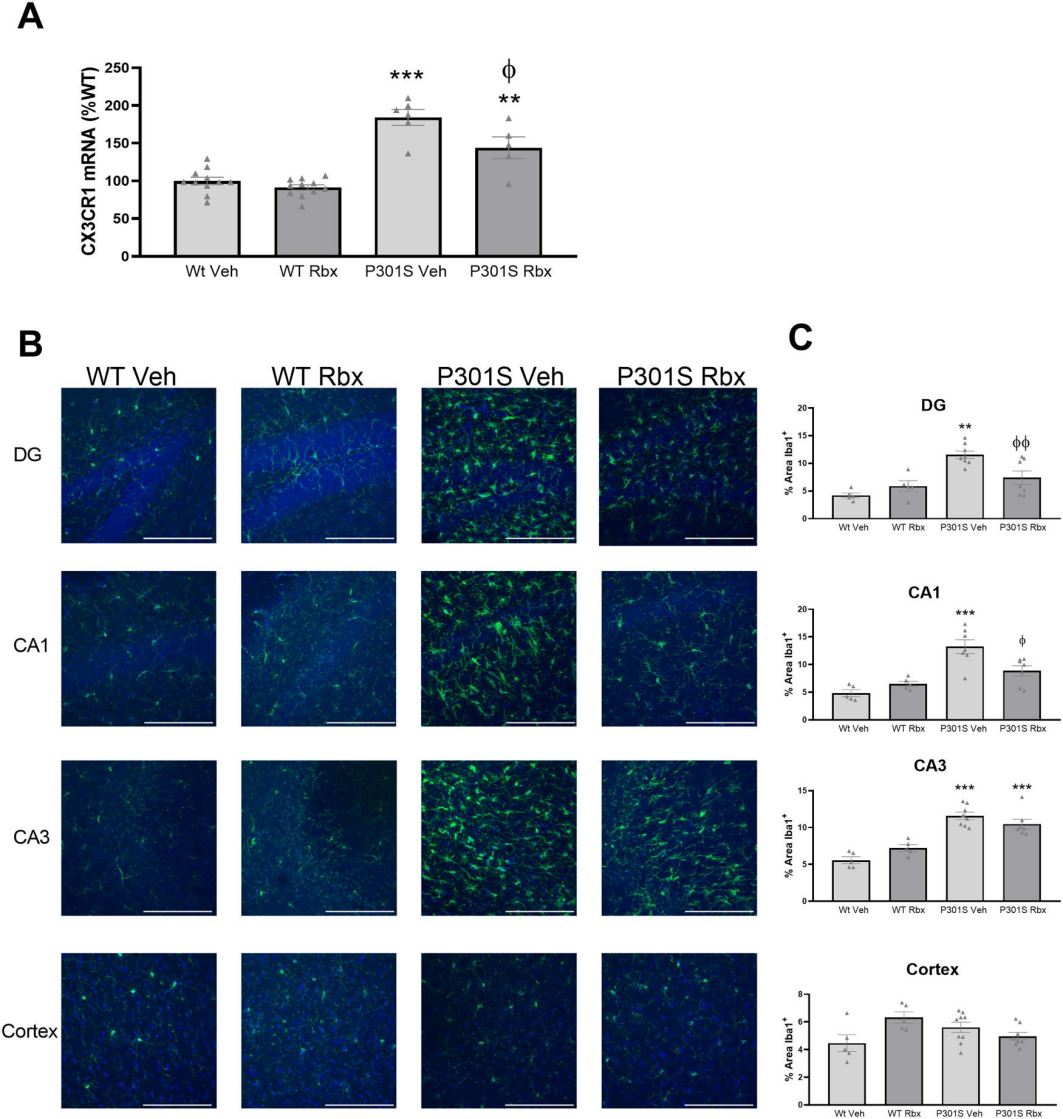
Reboxetine reduces microglial density in P301S mice hippocampus. (A) CX3CR1 mRNA concentrations were analyzed in hippocampus samples from WT and P301S mice treated with vehicle (Veh) or reboxetine (Rbx). Data are means ± SE of *n* ≥ 5 replicates per group. ***p < 0.001, **p < 0.01 vs. WT Veh. ^φ^*p* < 0.05 vs. P301S Veh. (B) Representative confocal image stacks of the dentate gyrus (DG), CA1, CA3 and frontal cortex (primary somatosensory area) from coronal sections prepared from WT and P301S mice treated with vehicle (Veh) or reboxetine (Rbx) stained for Iba1 (green) and DAPI (blue). Scale bar = 100μm. (C) Percentages of Iba1 positive immunoreactive areas. Data are means ± SE of *n* ≥ 5 replicates per group. ***p < 0.001, **p < 0.01 vs. WT Veh. ^φφ^*p* < 0.01, ^φ^*p* < 0.05 vs. P301S Veh.

The comparison of CX3CR1 mRNA levels in hippocampal samples indicates that the expression of this receptor is increased in P301S mice (Figure 1A). This expected alteration suggests the presence of a larger number of microglia cells in P301S mice hippocampi, in agreement with previous descriptions of this type of mice (Yoshiyama et al., 2007), as this receptor is almost exclusively expressed on microglial cells (Harrison et al., 1998). Interestingly, this increase was significantly lower in the P301S mice that were treated with reboxetine (Sidak’s multiple comparisons test, *t* = 3.33, *p* = 0.0141). According to this, reboxetine effects could result in an alteration of the microglial density characteristic of this model of tauopathy.

However, the PCR data may only reflect a mere increase in CX3CR1 mRNA without any alteration in the total number of microglia. Therefore, to confirm that reboxetine effect on CX3CR1 expression is associated to a reduction of microglial accumulation in the hippocampus, immunohistochemical analyses were performed using Iba1 as a marker of microglia. This way, as shown on Figure 1(B,C), all hippocampal areas analyzed presented a much larger number of stained cells in P301S mice in comparison to WT ones. Additionally, supporting CX3CR1 mRNA alterations observed, this technique allowed us to confirm that reboxetine treatment reduces the overall number of microglia seen in this model at the time point analyzed in the DG and CA1 areas of the hippocampus (Sidak’s multiple comparisons test, DG *t* = 3.51, *p* = 0.0123; CA1, *t* = 3.44, *p* = 0.0156).

On the other hand, no significant differences were observed in cortex samples. For this reason, based on our observations and those of others which have demonstrated that the hippocampus is known to be more susceptible to damage by tau (Aumont et al., 2025) in Alzheimer′s disease, our next analyses were focused on this brain area.

**Figure 2.**
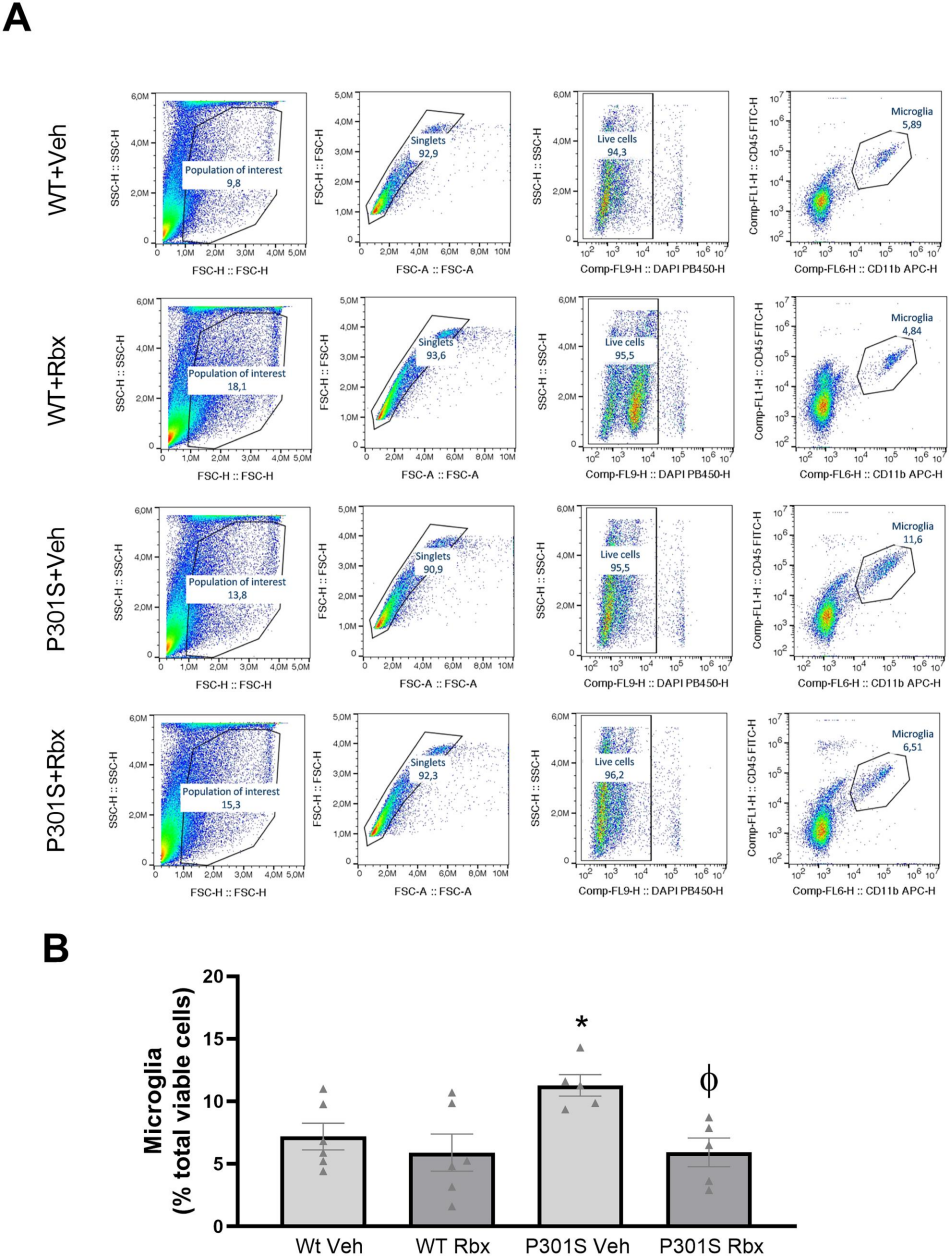
Flow cytometry analysis of reboxetine effect on microglial density in P301S mice hippocampus. (A) Representative dot plots showing the gating strategy used to distinguish microglial cells. (B) Percentages of microglia among the total number of cells present in hippocampal homogenates. Data are means ± SE of *n* ≥ 5 replicates per group. *p < 0.05 vs. WT Veh. ^φ^*p* < 0.05 vs. P301S Veh.

This way, flow cytometry tests were performed using hippocampal samples. These studies allowed us to achieve a more detailed evaluation, since the analyses comprised all the cells included in the hippocampus tissue isolated. As shown in Figure 2(B), the count of microglial cells demonstrates that, in comparison to WT mice, the number of microglia increases more than two-fold in P301S, while reboxetine treatment nearly halved this increase. Therefore, we can conclude that the administration of reboxetine prevents the increased accumulation of microglia that is observed in P301S mice (Sidak’s multiple comparisons test, *t* = 3.01, *p* = 0.043).

### Analysis of Reboxetine Effects on Microglial Morphology

Having confirmed by different means the effect of reboxetine on the accumulation of microglia in the hippocampus, we decided to examine the morphology of these cells to determine their homeostatic or reactive status.

**Figure 3.**
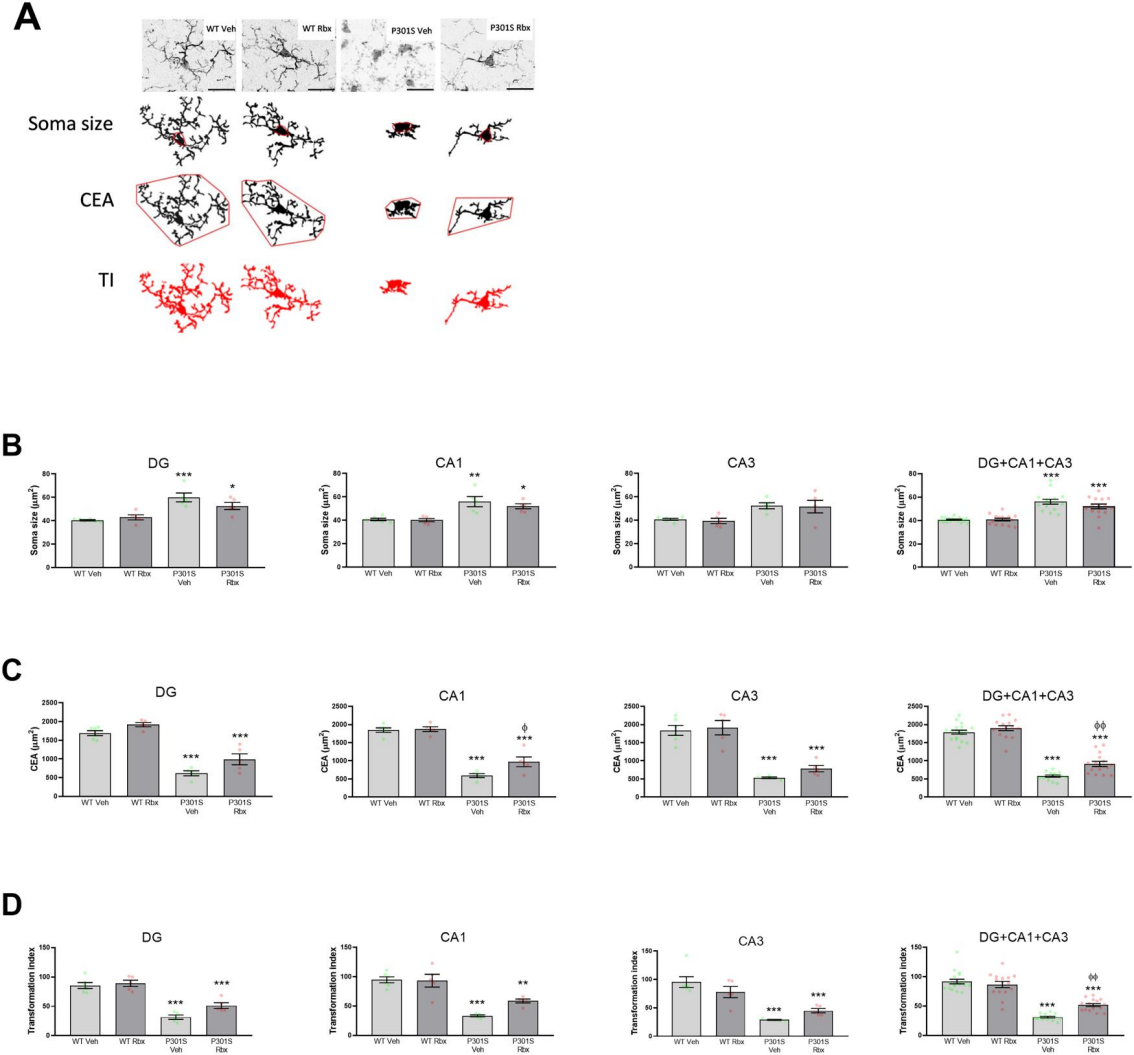
Microglial development of pro-inflammatory morphology is prevented by reboxetine treatment in P301S mice. (A) Representative images of Iba1^+^ cells used for morphological analyses (top) and cellular tracings (in red) depicting the characteristics analyzed through the measurement of soma size, covered environment area (CEA) and transformation index (TI). Scale bar = 20 μm. (B) Soma sizes, (C) CEAs and (D) TIs of microglia at DG, CA1, and CA3 separately and combined. Data are means ± SE of *n* ≥ 5 replicates per group. ***p < 0.001, **p < 0.01, *p < 0.05 vs. WT Veh. ^φφ^*p* < 0.01, ^φ^*p* < 0.05 vs. P301S Veh.

The quantification of the soma sizes allowed us to observe that, in all three hippocampal areas analyzed, microglia from P301S mice displayed larger cell bodies (Figure 3B). Once again, these differences, as expected, confirmed that the degree of microglial activation, known to be associated to an enlargement of the soma (Kozlowski & Weimer, 2012), is increased in P301S mice. Reboxetine-treated animals had a reduction of this parameter, which indicates the anti-inflammatory effect of the drug in the P301S model of tauopathy. But the changes were small, and no significant differences were detected even when the data obtained from DG, CA1 and CA3 areas were analyzed in combination.

While the transition from a resting or surveilling state of microglia to a reactive one is associated to alterations in the soma, it is also possible to characterize the different states by comparing the length and number of their projections. For this reason, further analyses were made in which these factors were quantified.

First, the covered environment area (CEA) was quantified for at least 45 cells in each hippocampal area. This parameter measures the total area occupied by the shape formed by linking all the extremities of a cell and is considered to be inversely proportional to the degree of microglial activation (Verdonk et al., 2016). The results obtained demonstrate that the average area covered by microglia from P301S mice is reduced in comparison to WT ones (Figure 3C). And more relevantly, reboxetine treatment largely prevented the reduction of CEA being this effect significant in the CA1 hippocampal area (Sidak’s multiple comparisons test, t = 3.142, p = 0.0352), and when all analyzed areas were combined (Sidak’s multiple comparisons test, t = 4.016, p = 0.0010).

Next, the transformation index (TI) was measured as an additional way to quantify the degree of activation in microglia. This index allows to evaluate the degree of ramification from a cell independently from its size, corresponding higher TI values to more ramified and less reactive cells. In agreement with the other two analyses performed, a reduction in the TI values was observed in P301S mice (Figure 3D), being this alteration prevented by reboxetine treatment (DG+CA1 + CA3, Sidak’s multiple comparisons test, *t* = 3.91, *p* = 0.0014).

### Reboxetine Treatment Reduces Astrocytic Density and Activation in P301S Mice Hippocampus

Once the effects of reboxetine on microglial activation were confirmed by the above-described studies, we decided to analyze if reboxetine actions also affect astrocytes. These cells, in combination with microglia, constitute the main agents responsible for the neuroinflammatory response (Han et al., 2025).

**Figure 4.**
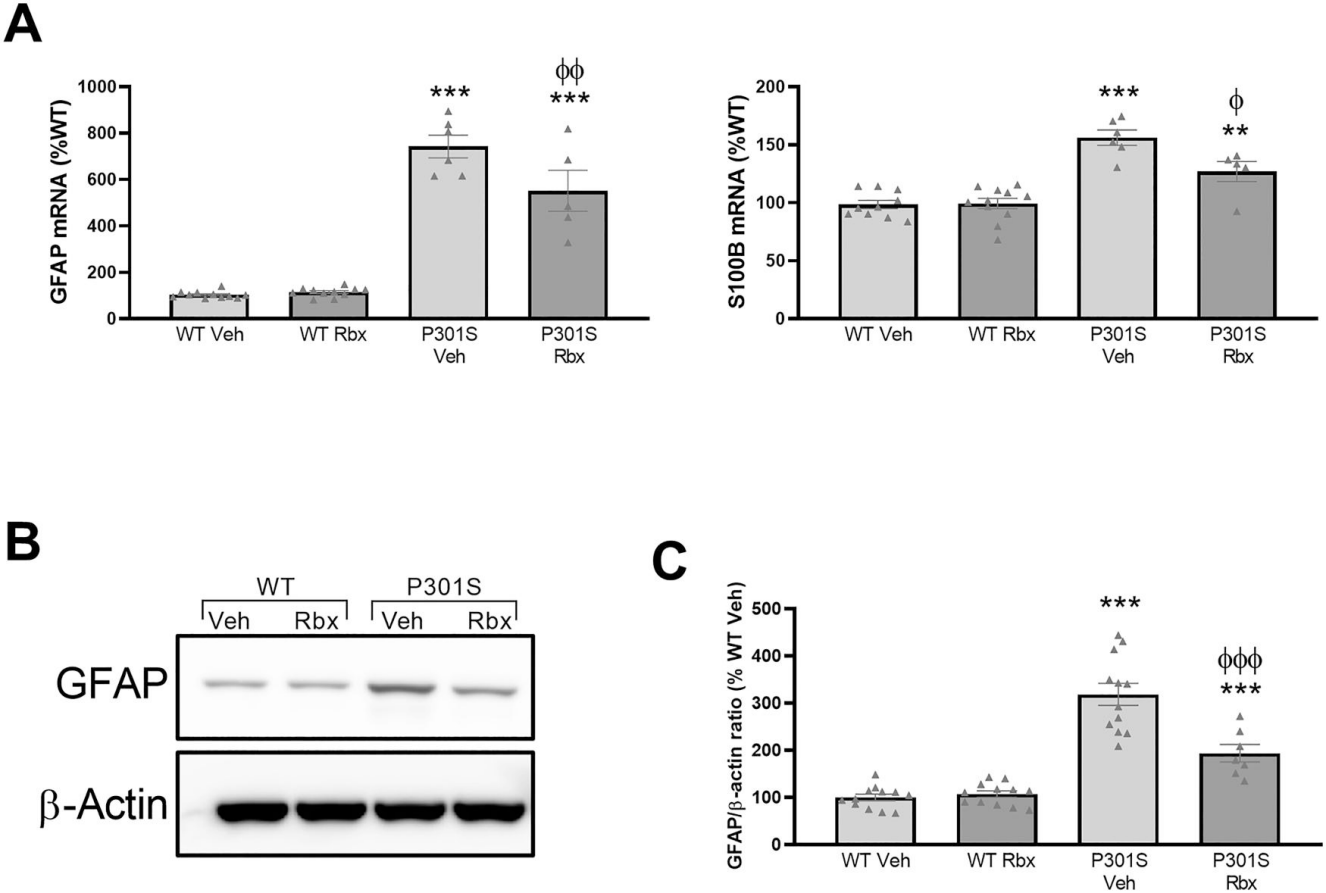
Reboxetine reduces the expression and synthesis of astrocyte markers in P301S mice hippocampus. (A) GFAP and S100B mRNA concentrations were analyzed in hippocampus samples from WT and P301S mice treated with vehicle (Veh) or reboxetine (Rbx). Data are means ± SE of *n* ≥ 5 replicates per group. ***p < 0.001, **p < 0.01 vs. WT Veh. ^φφ^*p* < 0.01, ^φ^*p* < 0.05 vs. P301S Veh. (B) Protein levels of GFAP and β-actin were analyzed by Western blot in hippocampal samples from WT and P301S mice treated with vehicle (Veh) or reboxetine (Rbx). The gels shown are representative of three separate experiments. (C) Densitometric analysis of the bands. AU: arbitrary units relative to WT Veh. Data are means ± SE of *n* ≥ 7 replicates per group. ***p < 0.001 vs. WT Veh. ^φφφ^*p* < 0.001, vs. P301S Veh.

For this, we first quantified the mRNA levels of two of the main astrocyte markers, glial fibrillary acidic protein (GFAP) and S100 calcium-binding protein B (S100B) in the hippocampus samples obtained from WT and P301S. The PCR results obtained (Figure 4A) indicate that the expression of *GFAP* and *S100B* is increased in P301S mice as expected. Interestingly, the expression of both markers was reduced in the samples obtained from reboxetine-treated mice (Sidak’s multiple comparisons test, GFAP: *t* = 3.516, *p* = 0.0087, S100B: *t* = 3.260, *p* < 0.0174), in a similar way as that observed in the microglial studies. In this case, mRNA differences between both mice genotypes (WT and P301S), as well as those between treatments (vehicle and reboxetine) were larger for *GFAP*.

To prove that the differences in mRNA levels detected are followed by similar ones in the synthesis of their corresponding proteins, the concentration of GFAP protein was analyzed by Western blot (Figure 4B). This way, as shown in Figure 4(C) we could confirm the differences between both types of mice and quantified the effect of reboxetine (Sidak’s multiple comparisons test, *t* = 5.220, *p* < 0.0001).

**Figure 5.**
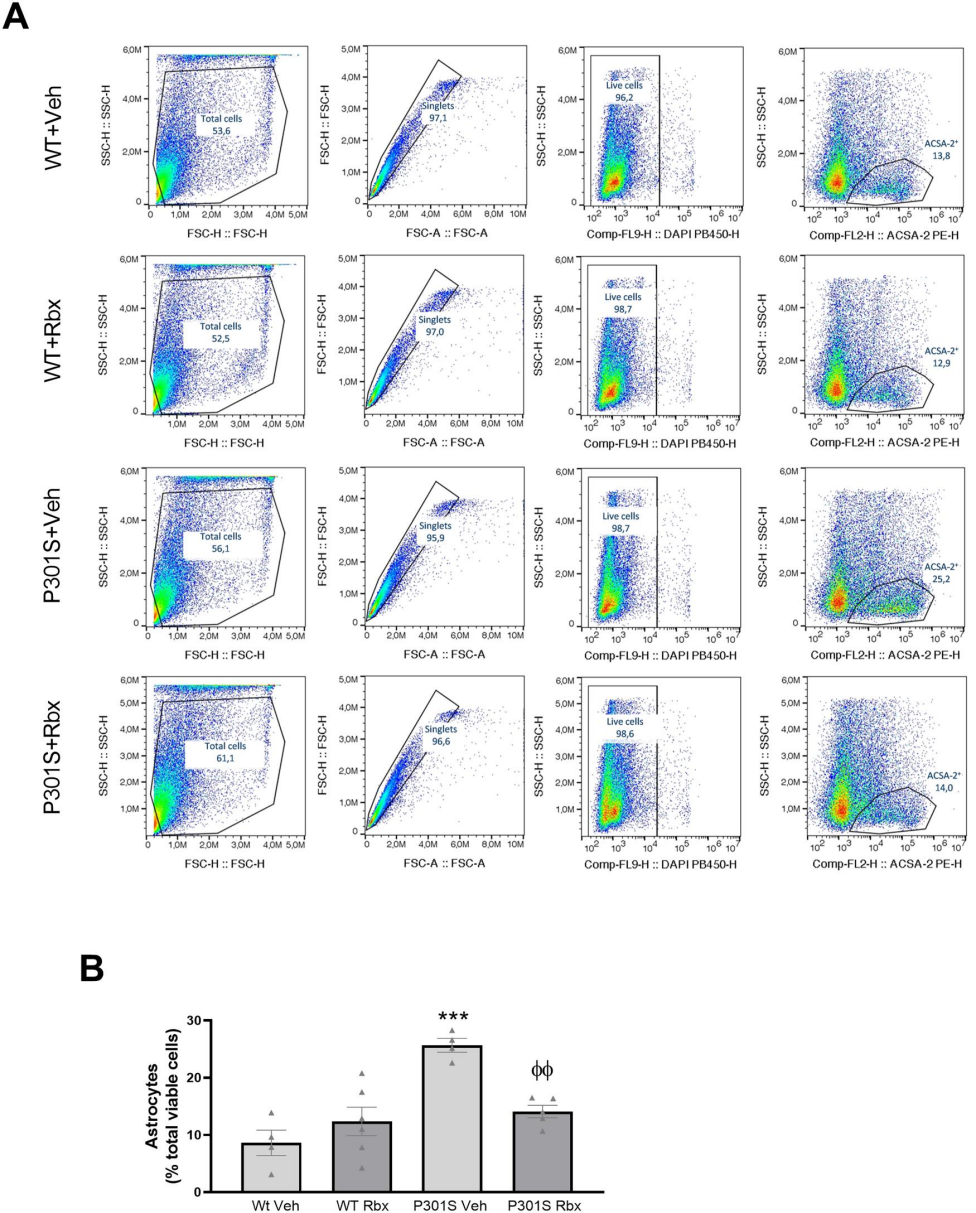
Flow cytometry analysis of reboxetine effect on astrocytic density in P301S mice hippocampus. (A) Representative dot plots showing the gating strategy used to distinguish astrocytes. Astrocytes were identified as ACSA-2^+^ cells. (B) Percentages of astrocytes among the total number of cells present in hippocampal homogenates. Data are means ± SE of *n* ≥ 4 replicates per group. ***p < 0.001 vs. WT Veh. ^φφ^*p* < 0.01 vs. P301S Veh.

Based on this, following a procedure similar to the one used for microglia, we performed flow cytometry analyses to further demonstrate the existence of different densities of astrocytes among our experimental groups (Figure 5). The data obtained strengthen PCR and Western blot results, and further demonstrate that reboxetine treatment prevents the accumulation of astrocytes in the hippocampus (Sidak’s multiple comparisons test, *t* = 3.942, *p* = 0.0078) that takes place in P301S mice.

### Analysis of Reboxetine Effects on Astrocytic Morphology

**Figure 6.**
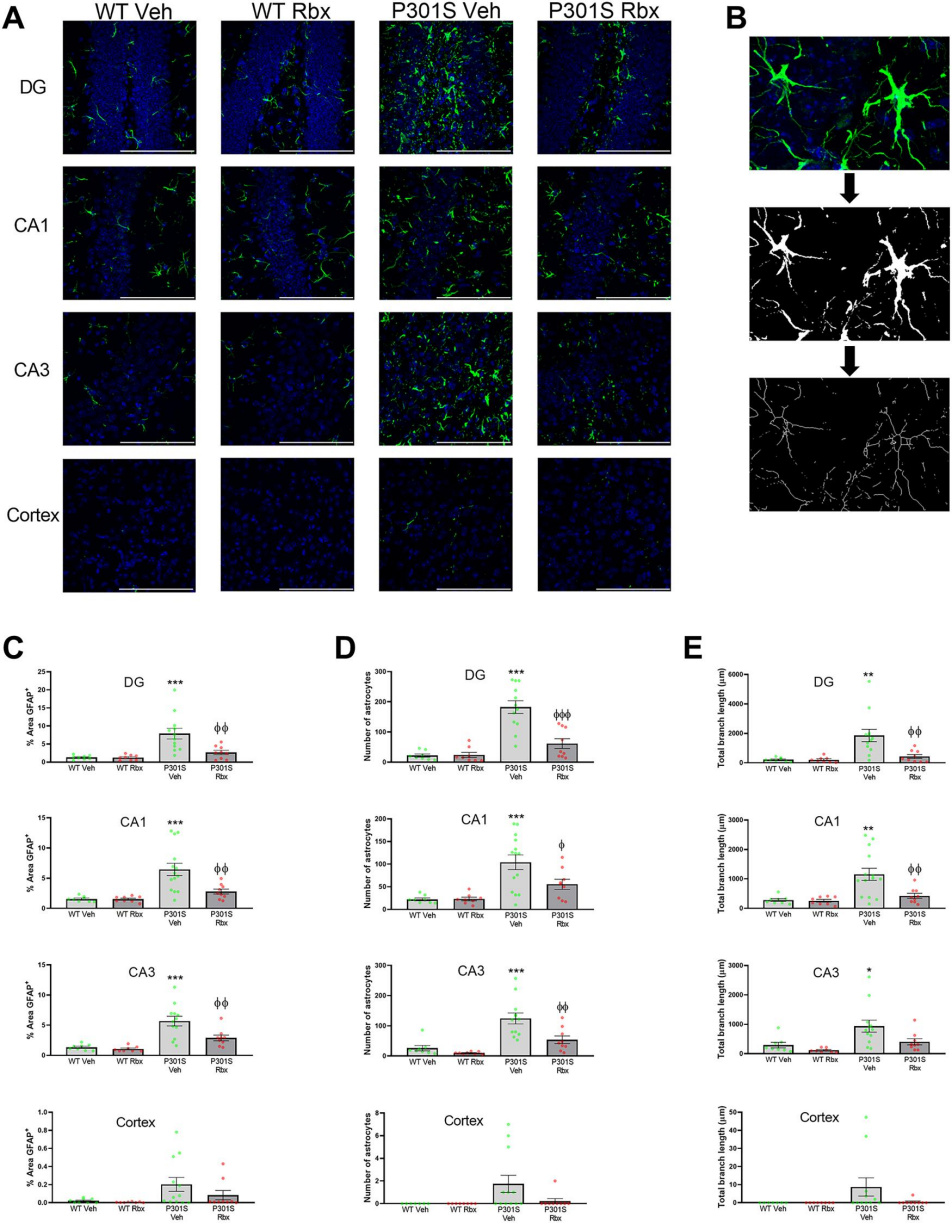
Reboxetine reduces astrocytes density and activation in P301S mice hippocampus. (A) Representative confocal image stacks of the dentate gyrus (DG), CA1, CA3, and frontal cortex (primary somatosensory area) from coronal sections prepared from WT and P301S mice treated with vehicle (Veh) or reboxetine (Rbx) stained for GFAP (green) and DAPI (blue). Scale bar = 100 μm. (B) Representative images depicting the process followed to measure the area, number of cells and branch length. (C) Percentages of GFAP positive immunoreactive areas. (D) Number of GFAP positive cells per field. (E) Total length of branches from GFAP positive cells per field. Data are means ± SE of *n* ≥ 8 replicates per group. ***p < 0.001, **p < 0.01, *p < 0.05 vs. WT Veh. ^φφ^*p* < 0.01, ^φ^*p* < 0.05 vs. P301S Veh.

Since PCR, Western blot and flow cytometry determinations for astrocytes were performed using whole hippocampi homogenates, additional immunohistochemical analyses were performed to obtain complementary data that allowed us to separately evaluate different hippocampus areas such as DG, CA1, and CA3. For this, brain slides were immunostained for GFAP and DAPI. As shown in Figure 6(A), the differences between genotypes and treatments could be noticed in all brain areas analyzed. Furthermore, the quantification of GFAP-labeled cells allowed us to confirm the effect of reboxetine on the number of astrocytes (Figure 6D) and on the total area occupied by these cells (Figure 6C). The differences between vehicle and reboxetine-treated P301S mice were statistically significant in all three hippocampal areas analyzed (Sidak’s multiple comparisons test: number of astrocytes (DG: *t* = 5.43, *p* < 0.0001, CA1: *t* = 2.792, *p* = 0.0495, CA3: *t* = 3.759, *p* = 0.004), area (DG: *t* = 3.794, *p* = 0.0036, CA1: *t* = 3.571, *p* = 0.0063, CA3: *t* = 3.560, *p* = 0.0069)).

While the density and size of astrocytes allow to assess the degree of neuroinflammation, another factor particularly useful for this purpose is the quantification of the extension of astrocytes branches. Therefore, following the methodology described by Marques et al. (Marques et al., 2023), the length of the astrocytic branches included in each stack of images was measured (Figure 6E). The results obtained using this methodology also demonstrate the increase of activated astrocytes, known to have larger branches (Pekny & Pekna, 2004), in P301S mice and the anti-inflammatory effect of reboxetine (Sidak’s multiple comparisons test: DG (*t* = 3.686, *p* = 0.0049), CA1 (*t* = 3.471, *p* = 0.0084), CA3 (*t* = 2.659, *p* = 0.0698)). However, no significant effect could be detected for reboxetine on CA3 area even though it reduced the length of astrocytic branches.

In addition, cortical areas were also included in the immunohistochemical analyses. As can be seen in Figure 6(D), the number of GFAP-labeled astrocytes was largely reduced in the cortex in comparison to the hippocampus. This difference is characteristic of P301S mice (Yoshiyama et al., 2007) and is similar to the reduced density of microglia we also found in the cortex (Figure 1B). In this case, however, while most of the samples analyzed had very low amounts of GFAP-labeled astrocytes, P301S mice showed a clear increase in the number of GFAP-labeled astrocytes, as well as in the area occupied by these cells and the length of their branches. The values detected for these parameters in reboxetine-treated P301S mice, although not statistically significant, were much lower. This suggests that, also in the cortex, reboxetine has a potent effect reducing the overall number of GFAP-labeled astrocytes seen in this model at the time point analyzed as well as their activation.

## Discussion

The results obtained in this study demonstrate that the administration of reboxetine to P301S mice reduces the accumulation of microglia and astrocytes in different parts of the hippocampus. Moreover, the degree of activation, determined through the analysis of the morphology of these cells, seems to be significantly reduced by the pharmacological treatment applied.

Therefore, in addition to the therapeutic indications already accepted for this drug, reboxetine may also have beneficial effects in AD and other pathologies involving tau dysfunctions and accumulation.

The lesions characteristic of P301S mice are primarily concentrated within the hippocampus (Yoshiyama et al., 2007). In fact, we could not detect large differences in other brain areas. But the quantification of GFAP-stained cells in the brain cortex suggests that, also in this region, reboxetine treatment prevents the astrogliosis characteristic of AD.

One of the main limitations in the search for new therapies to treat AD patients is the lack of a clear and well-defined target. This situation, common to many other life-threatening diseases, is due to the limited knowledge of AD. Despite the tremendous efforts done by diverse researchers and the spectacular advancements achieved in the last years, we are still far from a complete understanding of the causes and mechanisms involved in the development and progression of AD. This is confirmed by the small number of drugs approved for AD and the even smaller number of mechanisms of their actions, namely, the inhibition of acetylcholine degradation, antagonism of NMDA receptors and more recently, the elimination of amyloid-β proteins with specific antibodies (Fox et al., 2025).

As a result of this limitation, and due to the huge impact of AD in our society, there is a great interest in procuring additional treatments. Therefore, in addition to the ones already accepted, many other therapies are being tested. Some of these potential future therapeutic strategies are focused on new mechanisms of action, including vasculature alterations, proteostasis, glucose metabolism or gene therapies focused on *APOE* regulation, among others. Most of these treatments pursue the amendment of certain disfunctions specific of AD, but some others are directed against brain alterations that also take place in different neuronal pathologies. One of these alterations is neuroinflammation, which may be the most relevant one due to its clear neurotoxic effect and its involvement in numerous brain diseases that, besides AD, include other neurodegenerative ones such as Parkinson’s or Huntington’s disease (Zhang et al., 2023). Furthermore, neuroinflammation is also observed in deleterious conditions such as traumatic brain injuries (Kursancew et al., 2024), stroke, most neuropsychiatric conditions, including major depression and schizophrenia (Çakici et al., 2019) or different kinds of infections (Kettunen et al., 2024). For this reason, agents able to reduce the damage caused by neuroinflammation seem to be one of the most interesting tools worth studying due to their ability to reduce the loss of neurons in various central pathologies, even in cases, such as AD, in which the cause of damage may not be clearly identified.

One of the most interesting anti-inflammatory mediators in the central nervous system is noradrenaline. This catecholamine, in addition to its many roles as neurotransmitter, has also been demonstrated to prevent neuroinflammation and protect neurons against different kinds of injuries (Madrigal et al., 2005; 2006; 2009). The connection of noradrenaline and AD was initially stablished when a reduction in the LC volume was detected in brain samples obtained from AD patients (Bondareff et al., 1982). Since the LC constitutes the main source of noradrenaline within the central nervous system, the involvement of noradrenaline in the development of AD was proposed. This led to additional studies that allowed to confirm noradrenaline role in AD by either depleting noradrenaline through different means (Hammerschmidt et al., 2013; Heneka et al., 2002), or by increasing noradrenaline levels in AD models (Braun & Feinstein, 2019; Kalinin et al., 2012).

According to these observations, drugs able to enhance the effects of noradrenaline in the brain have been proposed as potential treatments in AD (David et al., 2022; Durcan et al., 2025). This way, we previously observed that reboxetine also reduced the neuroinflammation characteristic of AD in 5xFAD mice (Gutiérrez et al., 2019). These mice are also used as a model of AD but, unlike the P301S ones used here, they only reproduce the excessive production of amyloid-β present in AD (Oakley et al., 2006). The results obtained in that study, in combination with the ones presented here, demonstrate that the same protocol of reboxetine administration reduces the neuroinflammatory response elicited by both amyloid-β and by microtubule-associated protein tau (MAPT), the two main factors known to be responsible for AD neurodegeneration (Bloom, 2014). This is an additional advantage of reboxetine in comparison to other treatments aimed exclusively at one of the potential causes of neurodegeneration in AD. Furthermore, reboxetine has also demonstrated to be beneficial in a mouse model of another neurodegenerative condition such Parkinson’s disease (Kreiner et al., 2019). However, while reboxetine is known to modulate brain noradrenaline distribution, the effects observed in these studies could also be caused by other unknown actions of reboxetine independent of this neurotransmitter. Therefore, additional analyses based on the blockade of noradrenaline known mediators would allow to confirm the role of noradrenaline in reboxetine neuroprotective actions.

The mice used here were 9 months old at the onset of the treatment. At this age, P301S mice present a degree of neuroinflammation and cognitive decline comparable to those observed in advanced cases of AD in humans (Yoshiyama et al., 2007). Similarly, the 5xFAD mice used in our previous study received the reboxetine treatment at an age in which their brain damage was at an advanced point (Oakley et al., 2006). Therefore, it can be concluded that reboxetine can exert its protective effects even if administered at advanced stages of AD. This increases the therapeutic relevance of reboxetine in comparison to some currently approved treatments which are only effective in early AD.

Nevertheless, the use of adequate conditions seems to be a relevant factor when administering reboxetine. This was observed in a study in which the effect of reboxetine was analyzed in mice obtained by crossing 5xFAD and P301L mice (Jeong et al., 2025). As shown in this publication, reboxetine treatment induces the aggregation of tau and neurological damage. These harmful effects seem to be caused by the excessive amounts of reboxetine administered over a relatively long period of time. As indicated by the authors, exposure to excessive noradrenaline may accelerate tau pathology instead of preventing it. According to this, great attention should be paid when considering the use of noradrenaline modulating drugs in neurodegenerative disorders due to the elevated risk of overdosing them. In fact, a similar study using even higher doses of reboxetine confirmed this threat of increasing tau toxicity (Koppel et al., 2019).

This potentiation of tau toxicity has been attributed to 3,4-dihydroxyphenylglycolaldehyde (DOPEGAL), a monoamine oxidase A metabolite of noradrenaline which has been shown to interact with tau stimulating its aggregation and propagation (Kang et al., 2020). Regardless of this, clinical tests have shown that another noradrenaline reuptake inhibitor such as atomoxetine reduces tau levels in the cerebrospinal fluid obtained from AD patients (Levey et al., 2022).

The duality of noradrenaline actions may also depend on the activation status of glial cells, as our previous analyses performed on cultured astrocytes demonstrate. These studies indicate that, in the presence of an inflammatory stimulus, noradrenaline reduces the activation and production of pro-inflammatory mediators, while it has the opposite effect in basal conditions (Hinojosa et al., 2013). This adds another factor, such as the existence of neuroinflammation, to consider when administering reboxetine or other noradrenaline modulating agent to reduce neurotoxicity.

However, since only male mice were used in this study, it is important to be cautious when interpreting the results obtained and their relevance for humans. Additional analyses should be performed to confirm the existence of similar effects of reboxetine in females before further advancement in the study of the therapeutic potential of reboxetine in AD. Additionally, the measurement of brain noradrenaline levels would allow to determine the degree of the reboxetine treatment effect at this level.

In summary, this study demonstrates that continuous administration of reboxetine for 28 days to aged MAPT-overexpressing mice reduces the activation of both microglia and astrocytes in their hippocampus. This supports the potential repurposing of reboxetine as a new treatment for AD.

Future AD treatments will probably consist of a combination of different treatments aimed at more than one single target. However, previous experiences demonstrate that, despite hopeful preliminary results, many of the clinical trials on progress may not result in the approval of new treatments as soon as desired.

In the meanwhile, the repurposing of drugs currently approved and extensively tested in humans over the last years, constitutes a promising strategy to reduce the time needed to offer new AD treatments.

## Supplementary Material

ANOVA data.docx

Supplemental figure 1 REVISED.pdf

## Data Availability

The data that support the findings of this study are available from the corresponding author upon reasonable request.
