# Supplementary material for: Reboxetine Treatment Reduces Hippocampal Gliosis in the P301S Tauopathy Mouse Model
Source: ASN Neuro. 2026 Feb 21;18(1):2630485. doi: 10.1080/17590914.2026.2630485 (PMC12928618; doi:10.1080/17590914.2026.2630485)
Supplement: ANOVA data.docx [file TASN_A_2630485_SM6692.docx]

**ANOVA RESULTS**

**Figure 1A**

Genotype: F_(1, 29)_=85.83, p<0.0001

Treatment: F_(1, 29)_=10.98, p=0.0025

Interaction: F_(1, 29)_=4.566, p=0.0412

**Figure 1C**

DG

Genotype: F_(1, 21)_=22.95, p<0.0001

Treatment: F_(1, 21)_=1.73, p=0.20

Interaction: F_(1, 21)_=9.828, p=0.0050

CA1

Genotype: F_(1, 20)_=29.87, p<0.0001

Treatment: F_(1, 20)_=1.86, p=0.18

Interaction: F_(1, 20)_=9.435, p=0.0060

CA3

Genotype: F_(1, 21)_=68.49, p<0.0001

Treatment: F_(1, 21)_=0.23, p=0.63

Interaction: F_(1, 21)_=6.036, p=0.0228

Cortex

Genotype: F_(1, 23)_=0.07206, p=0.7908

Treatment: F_(1, 23)_=2.186, p=0.1529

Interaction: F_(1, 23)_=9.155, p=0.0060

**Figure 2B**

Genotype: F_(1, 18)_=2.928, p=0.1042

Treatment: F_(1, 18)_=7.630, p=0.0128

Interaction: F_(1, 18)_=2.859, p=0.1081

**Figure 3B**

DG

Genotype: F_(1, 17)_=32.62, p<0.0001

Treatment: F_(1, 17)_=0.908, p=0.354

Interaction: F_(1, 17)_=3.686, p=0.0718

CA1

Genotype: F_(1, 17)_=30.49, p<0.0001

Treatment: F_(1, 17)_=0.838, p=0.372

Interaction: F_(1, 17)_=0.513, p=0.4832

CA3

Genotype: F_(1, 17)_=15.20, p=0.0012

Treatment: F_(1, 17)_=0.119, p=0.734

Interaction: F_(1, 17)_=0.007, p=0.932

DG+CA1+CA3

Genotype: F_(1, 59)_=77.74, p<0.0001

Treatment: F_(1, 59)_=1.595, p=0.211

Interaction: F_(1, 59)_=1.986, p=0.164

**Figure 3C**

DG

Genotype: F_(1, 17)_=127.3, p<0.0001

Treatment: F_(1, 17)_=11.44, p=0.003

Interaction: F_(1, 17)_=0.680, p=0.420

CA1

Genotype: F_(1, 17)_=166.8, p<0.0001

Treatment: F_(1, 17)_=5.946, p=0.02

Interaction: F_(1, 17)_=4.410, p=0.051

CA3

Genotype: F_(1, 17)_=86.11, p<0.0001

Treatment: F_(1, 17)_=1.55, p=0.23

Interaction: F_(1, 17)_=0.463, p=0.505

DG+CA1+CA3

Genotype: F_(1, 59)_=361.9, p<0.0001

Treatment: F_(1, 59)_=14.83, p=0.0003

Interaction: F_(1, 59)_=3.803, p=0.0559

**Figure 3D**

DG

Genotype: F_(1, 17)_=89.34, p<0.0001

Treatment: F_(1, 17)_=5.80, p=0.02

Interaction: F_(1, 17)_=2.676, p=0.120

CA1

Genotype: F_(1, 17)_=59.3, p<0.0001

Treatment: F_(1, 17)_=3.68, p=0.07

Interaction: F_(1, 17)_=4.662, p=0.045

CA3

Genotype: F_(1, 17)_=44.41, p<0.0001

Treatment: F_(1, 17)_=0.009, p=0.92

Interaction: F_(1, 17)_=5.146, p=0.036

DG+CA1+CA3

Genotype: F_(1, 59)_=176.0, p<0.0001

Treatment: F_(1, 59)_=4.492, p=0.038

Interaction: F_(1, 59)_=12.52, p=0.0008

**Figure 4A**

GFAP

Genotype: F_(1, 29)_=260.7, p<0.0001

Treatment: F_(1, 29)_=7.30, p=0.011

Interaction: F_(1, 29)_=9.275, p=0.0049

S100B

Genotype: F_(1, 28)_=59.05, p<0.0001

Treatment: F_(1, 28)_=6.637, p=0.015

Interaction: F_(1, 28)_=7.352, p=0.011

**Figure 4C**

Genotype: F_(1, 39)_=93.66, p<0.0001

Treatment: F_(1, 39)_=13.94, p=0.0006

Interaction: F_(1, 39)_=17.54, p=0.0002

**Figure 5B**

Genotype: F_(1, 15)_=21.18, p=0.0003

Treatment: F_(1, 15)_=3.691, p=0.073

Interaction: F_(1, 15)_=14,14, p=0.001

**Figure 6C**

DG

Genotype: F_(1, 33)_=14.58, p=0.0006

Treatment: F_(1, 33)_=6.406, p=0.016

Interaction: F_(1, 33)_=6.190, p=0.018

CA1

Genotype: F_(1, 35)_=15.22, p=0.0004

Treatment: F_(1, 35)_=5.472, p=0.0252

Interaction: F_(1, 35)_=5.291, p=0.027

CA3

Genotype: F_(1, 33)_=27.49, p<0.0001

Treatment: F_(1, 33)_=6.752, p=0.013

Interaction: F_(1, 33)_=4.457, p=0.042

Cortex

Genotype: F_(1, 33)_=5.160, p=0.029

Treatment: F_(1, 33)_=1.383, p=0.248

Interaction: F_(1, 33)_=0.809, p=0.374

**Figure 6D**

DG

Genotype: F_(1, 33)_=34.75, p<0.0001

Treatment: F_(1, 33)_=12.45, p=0.0013

Interaction: F_(1, 33)_=13.36, p=0.0009

CA1

Genotype: F_(1, 35)_=18.27, p=0.0001

Treatment: F_(1, 35)_=3.174, p=0.083

Interaction: F_(1, 35)_=3.410, p=0.073

CA3

Genotype: F_(1, 33)_=24.82, p<0.0001

Treatment: F_(1, 33)_=9.29, p=0.004

Interaction: F_(1, 33)_=3.705, p=0.062

Cortex

Genotype: F_(1, 32)_=3.374, p=0.075

Treatment: F_(1, 32)_=2.024, p=0.164

Interaction: F_(1, 32)_=2.024, p=0.164

**Figure 6E**

DG

Genotype: F_(1, 33)_=10.27, p=0.003

Treatment: F_(1, 33)_=6.027, p=0.019

Interaction: F_(1, 33)_=5.861, p=0.021

CA1

Genotype: F_(1, 35)_=10.38, p=0.0028

Treatment: F_(1, 35)_=5.396, p=0.026

Interaction: F_(1, 35)_=4.780, p=0.035

CA3

Genotype: F_(1, 33)_=9.411, p=0.0043

Treatment: F_(1, 33)_=5.484, p=0.025

Interaction: F_(1, 33)_=1.383, p=0.248

Cortex

Genotype: F_(1, 31)_=1.979, p=0.169

Treatment: F_(1, 31)_=1.594, p=0.216

Interaction: F_(1, 31)_=1.594, p=0.216
