# Supplementary figures and images for: Reboxetine Treatment Reduces Hippocampal Gliosis in the P301S Tauopathy Mouse Model
Source: ASN Neuro. 2026 Feb 21;18(1):2630485. doi: 10.1080/17590914.2026.2630485 (PMC12928618; doi:10.1080/17590914.2026.2630485)

## GFAP

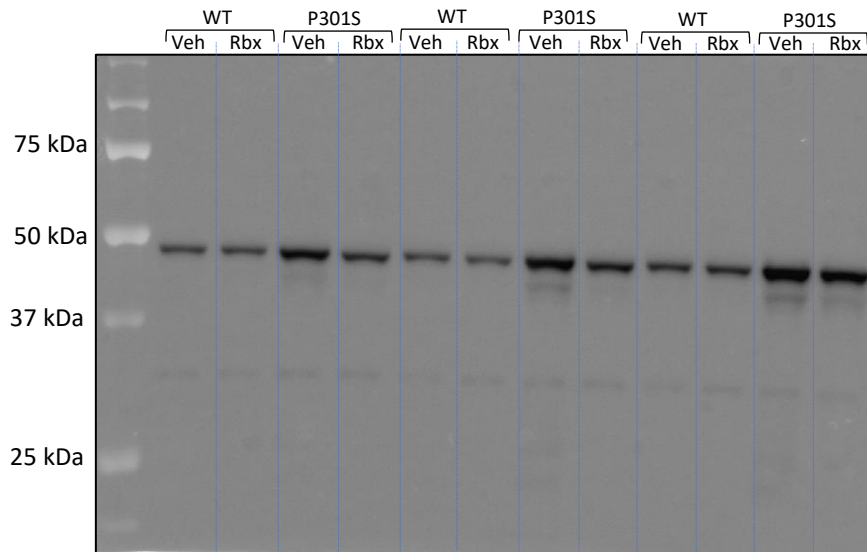

β-Actin

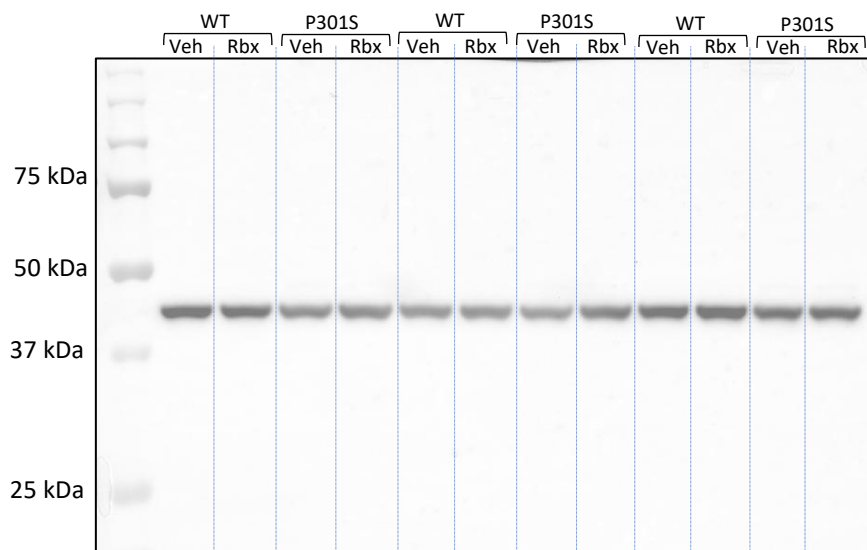

Supplement: Supplemental figure 1 REVISED.pdf [file TASN_A_2630485_SM6691.pdf]
